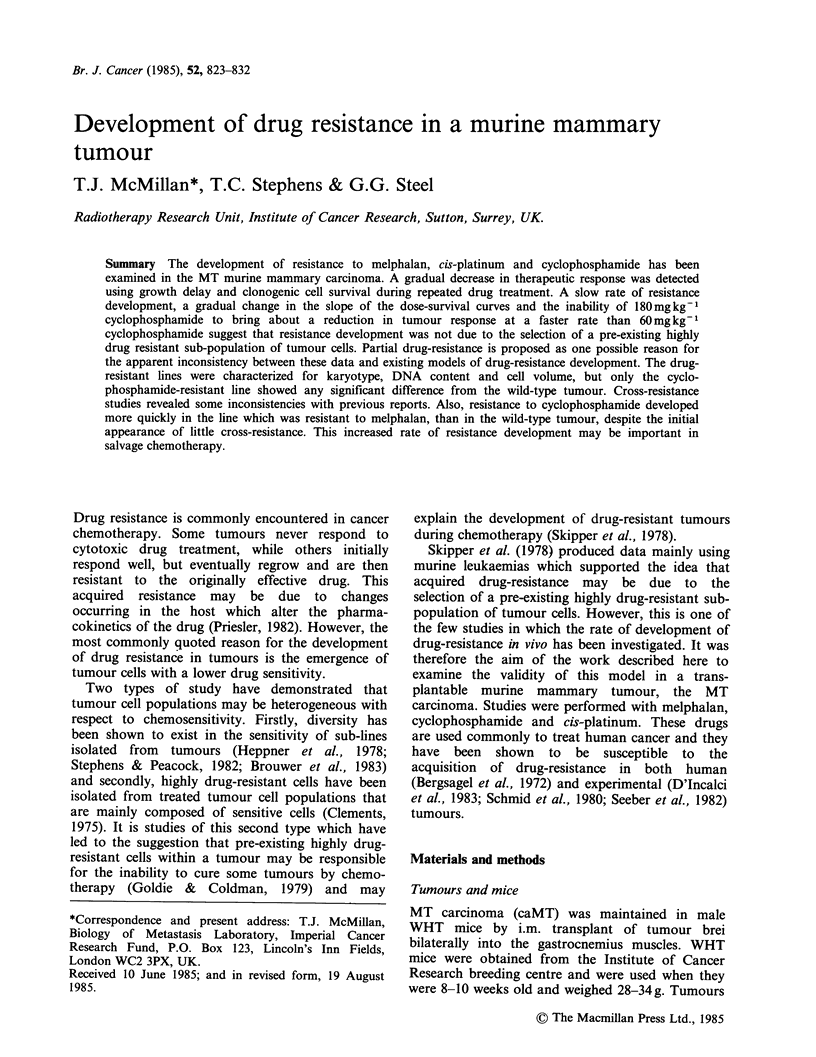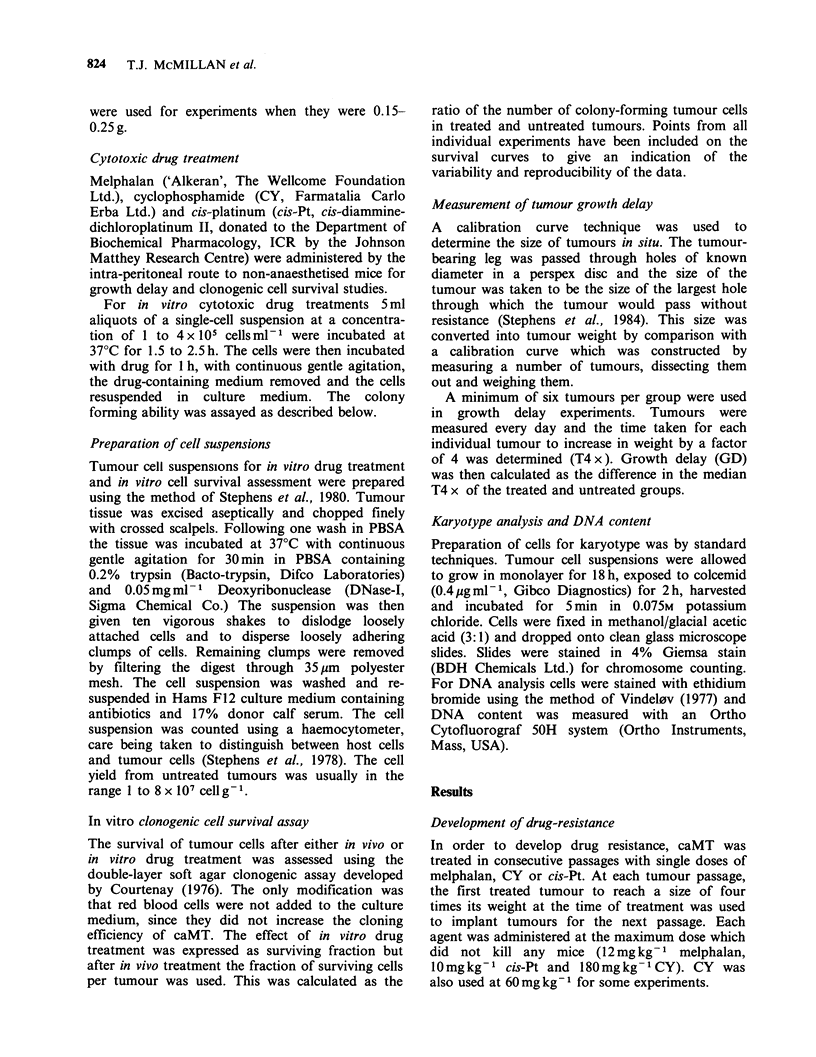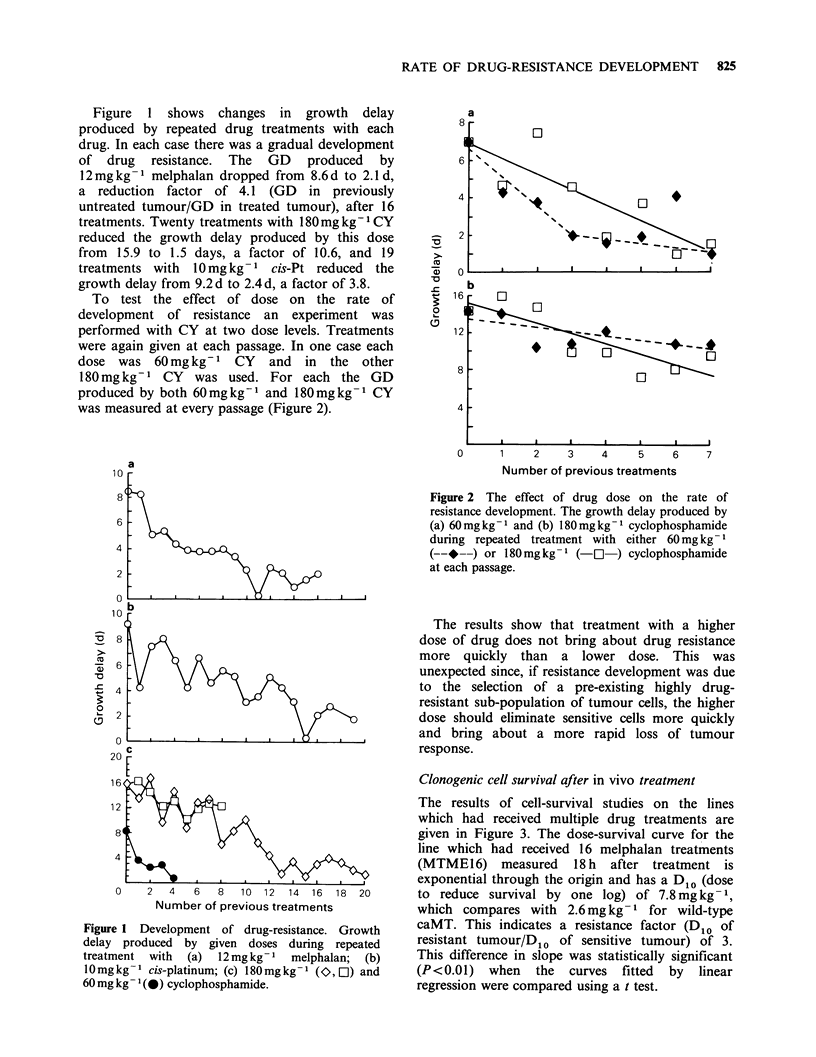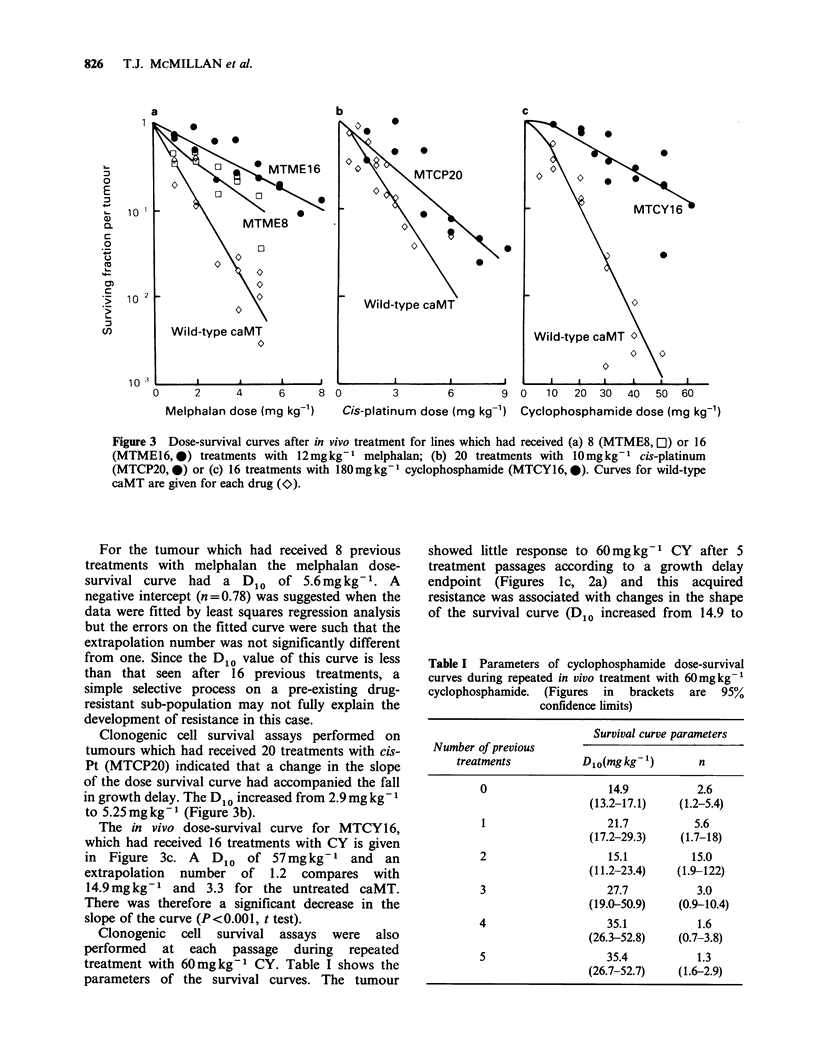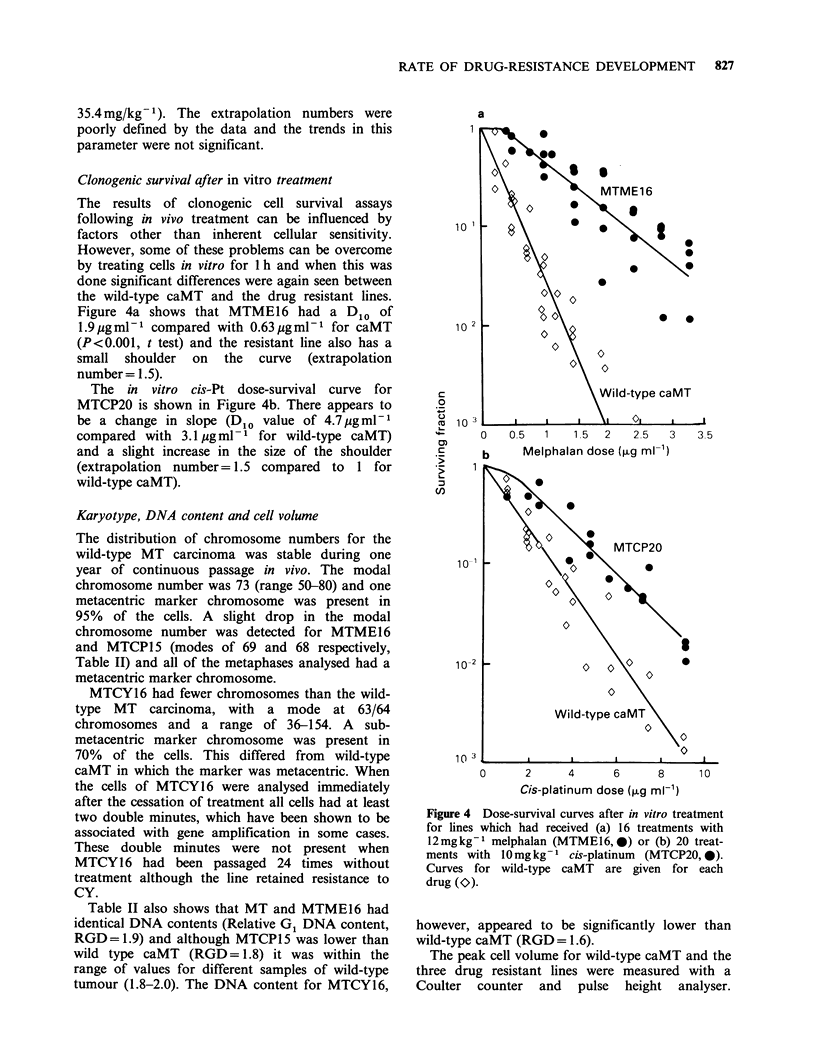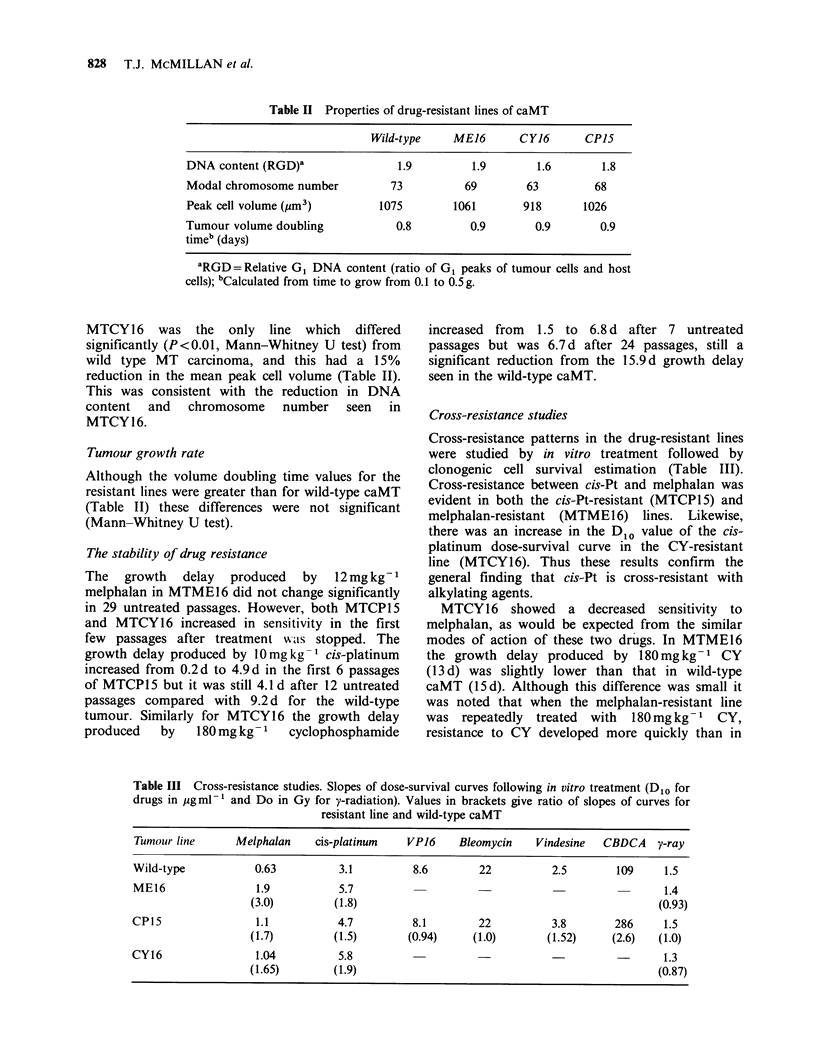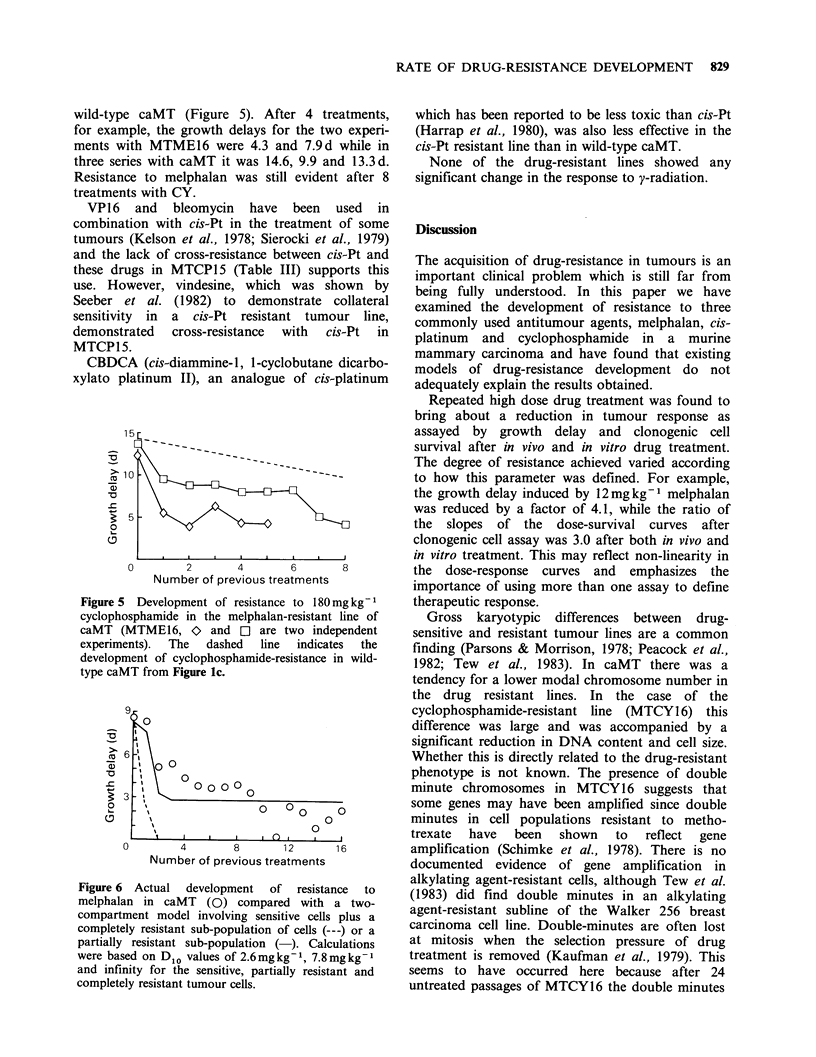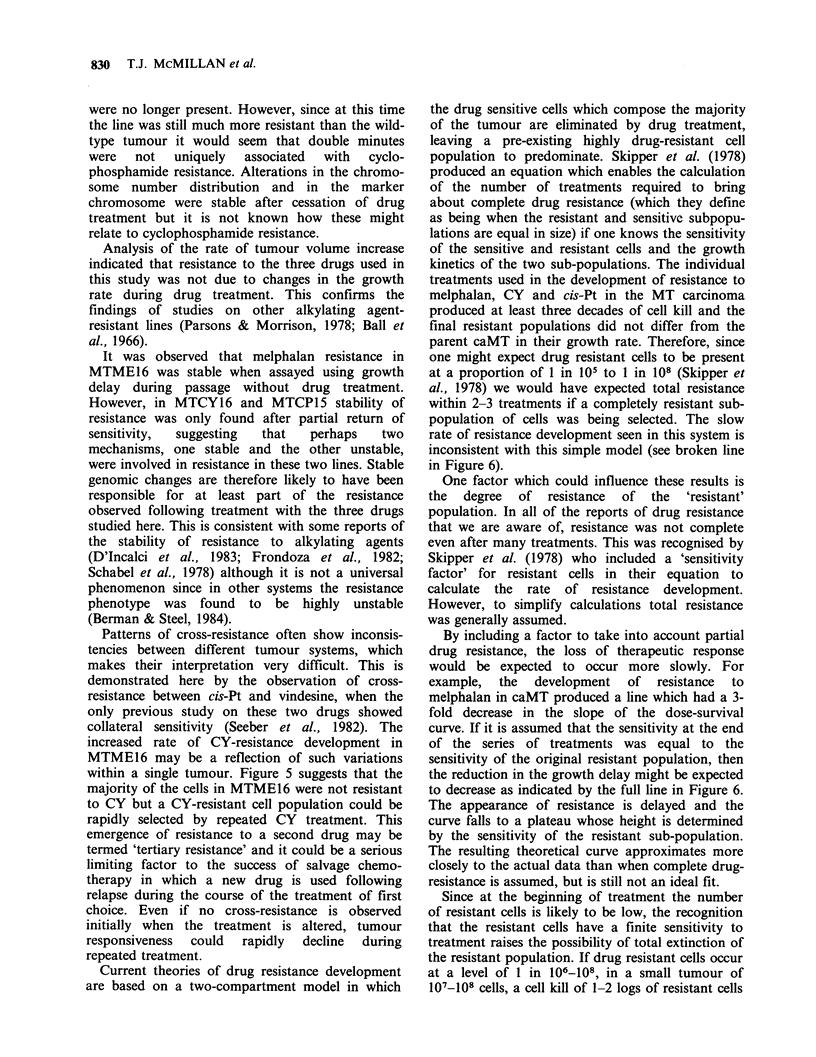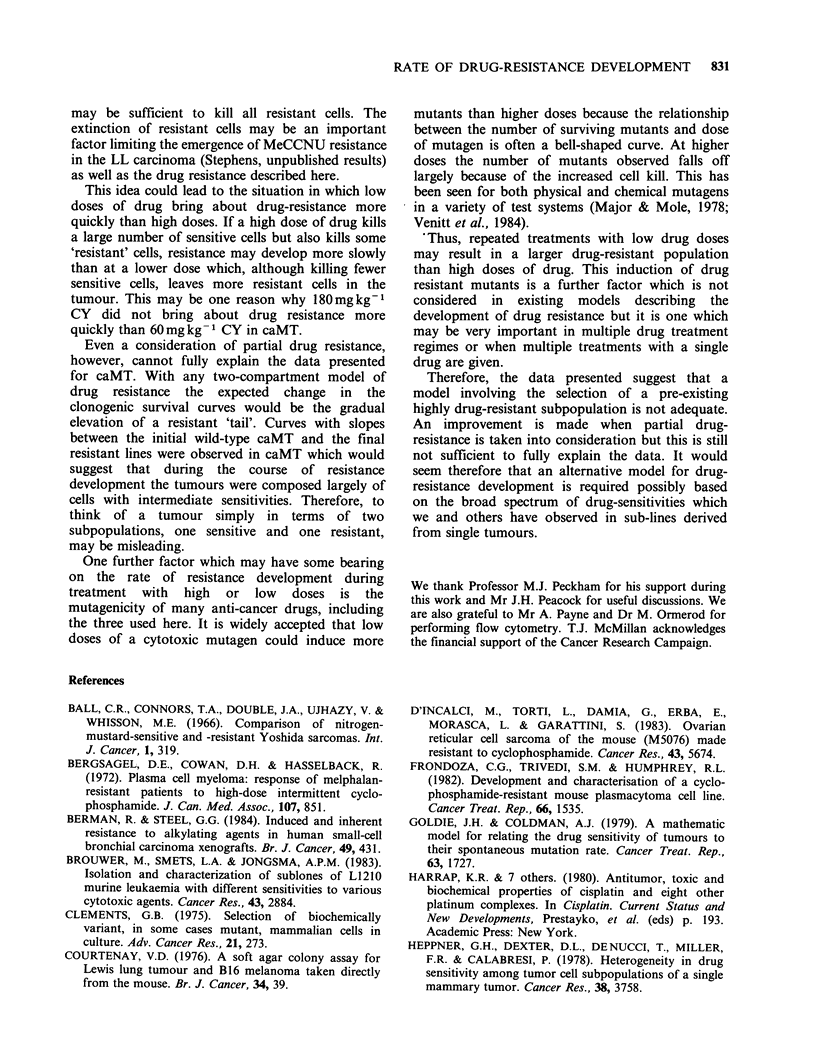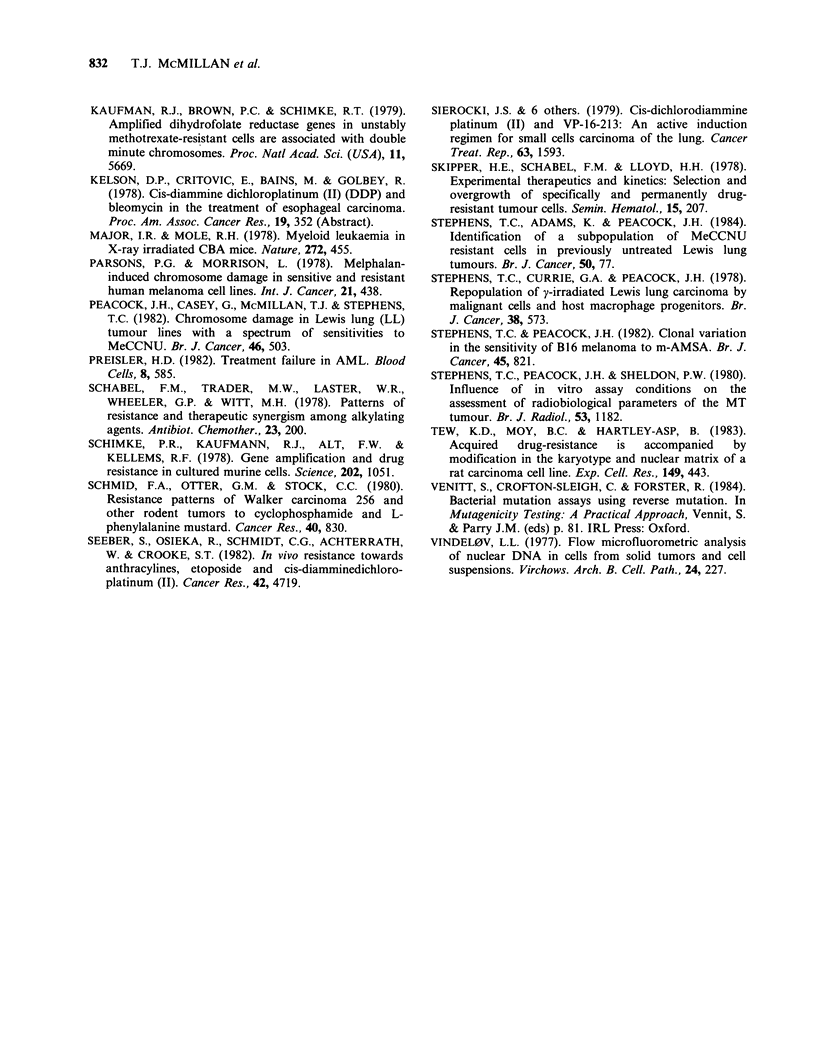# Development of drug resistance in a murine mammary tumour.

**DOI:** 10.1038/bjc.1985.265

**Published:** 1985-12

**Authors:** T. J. McMillan, T. C. Stephens, G. G. Steel

## Abstract

The development of resistance to melphalan, cis-platinum and cyclophosphamide has been examined in the MT murine mammary carcinoma. A gradual decrease in therapeutic response was detected using growth delay and clonogenic cell survival during repeated drug treatment. A slow rate of resistance development, a gradual change in the slope of the dose-survival curves and the inability of 180 mg kg-1 cyclophosphamide to bring about a reduction in tumour response at a faster rate than 60 mg kg-1 cyclophosphamide suggest that resistance development was not due to the selection of a pre-existing highly drug resistant sub-population of tumour cells. Partial drug-resistance is proposed as one possible reason for the apparent inconsistency between these data and existing models of drug-resistance development. The drug-resistant lines were characterized for karyotype, DNA content and cell volume, but only the cyclophosphamide-resistant line showed any significant difference from the wild-type tumour. Cross-resistance studies revealed some inconsistencies with previous reports. Also, resistance to cyclophosphamide developed more quickly in the line which was resistant to melphalan, than in the wild-type tumour, despite the initial appearance of little cross-resistance. This increased rate of resistance development may be important in salvage chemotherapy.


*Br. J. Cancer* (1985), **52**, 823–832

### Development of drug resistance in a murine mammary tumour

T.J. McMillan*, T.C. Stephens & G.G. Steel

*Radiotherapy Research Unit, Institute of Cancer Research, Sutton, Surrey, UK.*

**Summary** The development of resistance to melphalan, *cis*-platinum and cyclophosphamide has been examined in the MT murine mammary carcinoma. A gradual decrease in therapeutic response was detected using growth delay and clonogenic cell survival during repeated drug treatment. A slow rate of resistance development, a gradual change in the slope of the dose-survival curves and the inability of $180\,mg\,kg^{-1}$ cyclophosphamide to bring about a reduction in tumour response at a faster rate than $60\,mg\,kg^{-1}$ cyclophosphamide suggest that resistance development was not due to the selection of a pre-existing highly drug resistant sub-population of tumour cells. Partial drug-resistance is proposed as one possible reason for the apparent inconsistency between these data and existing models of drug-resistance development. The drug-resistant lines were characterized for karyotype, DNA content and cell volume, but only the cyclophosphamide-resistant line showed any significant difference from the wild-type tumour. Cross-resistance studies revealed some inconsistencies with previous reports. Also, resistance to cyclophosphamide developed more quickly in the line which was resistant to melphalan, than in the wild-type tumour, despite the initial appearance of little cross-resistance. This increased rate of resistance development may be important in salvage chemotherapy.

Drug resistance is commonly encountered in cancer chemotherapy. Some tumours never respond to cytotoxic drug treatment, while others initially respond well, but eventually regrow and are then resistant to the originally effective drug. This acquired resistance may be due to changes occurring in the host which alter the pharmacokinetics of the drug (Priesler, 1982). However, the most commonly quoted reason for the development of drug resistance in tumours is the emergence of tumour cells with a lower drug sensitivity.

Two types of study have demonstrated that tumour cell populations may be heterogeneous with respect to chemosensitivity. Firstly, diversity has been shown to exist in the sensitivity of sub-lines isolated from tumours (Heppner *et al.*, 1978; Stephens & Peacock, 1982; Brouwer *et al.*, 1983) and secondly, highly drug-resistant cells have been isolated from treated tumour cell populations that are mainly composed of sensitive cells (Clements, 1975). It is studies of this second type which have led to the suggestion that pre-existing highly drug-resistant cells within a tumour may be responsible for the inability to cure some tumours by chemotherapy (Goldie & Coldman, 1979) and may explain the development of drug-resistant tumours during chemotherapy (Skipper *et al.*, 1978).

Skipper *et al.* (1978) produced data mainly using murine leukaemias which supported the idea that acquired drug-resistance may be due to the selection of a pre-existing highly drug-resistant sub-population of tumour cells. However, this is one of the few studies in which the rate of development of drug-resistance *in vivo* has been investigated. It was therefore the aim of the work described here to examine the validity of this model in a transplantable murine mammary tumour, the MT carcinoma. Studies were performed with melphalan, cyclophosphamide and *cis*-platinum. These drugs are used commonly to treat human cancer and they have been shown to be susceptible to the acquisition of drug-resistance in both human (Bergsagel *et al.*, 1972) and experimental (D'Incalci *et al.*, 1983; Schmid *et al.*, 1980; Seeber *et al.*, 1982) tumours.

### Materials and methods

### *Tumours and mice*

MT carcinoma (caMT) was maintained in male WHT mice by i.m. transplant of tumour brei bilaterally into the gastrocnemius muscles. WHT mice were obtained from the Institute of Cancer Research breeding centre and were used when they were 8–10 weeks old and weighed 28–34 g. Tumours

*Correspondence and present address: T.J. McMillan, Biology of Metastasis Laboratory, Imperial Cancer Research Fund, P.O. Box 123, Lincoln's Inn Fields, London WC2 3PX, UK.

Received 10 June 1985; and in revised form, 19 August 1985.

© The Macmillan Press Ltd., 1985

were used for experiments when they were 0.15–0.25 g.

*Cytotoxic drug treatment*

Melphalan ('Alkeran', The Wellcome Foundation Ltd.), cyclophosphamide (CY, Farmatalia Carlo Erba Ltd.) and *cis*-platinum (*cis*-Pt, *cis*-diamminedichloroplatinum II, donated to the Department of Biochemical Pharmacology, ICR by the Johnson Matthey Research Centre) were administered by the intra-peritoneal route to non-anaesthetised mice for growth delay and clonogenic cell survival studies.

For *in vitro* cytotoxic drug treatments 5 ml aliquots of a single-cell suspension at a concentration of 1 to $4\times10^5$ cells $ml^{-1}$ were incubated at 37°C for 1.5 to 2.5 h. The cells were then incubated with drug for 1 h, with continuous gentle agitation, the drug-containing medium removed and the cells resuspended in culture medium. The colony forming ability was assayed as described below.

*Preparation of cell suspensions*

Tumour cell suspensions for *in vitro* drug treatment and *in vitro* cell survival assessment were prepared using the method of Stephens *et al.*, 1980. Tumour tissue was excised aseptically and chopped finely with crossed scalpels. Following one wash in PBSA the tissue was incubated at 37°C with continuous gentle agitation for 30 min in PBSA containing 0.2% trypsin (Bacto-trypsin, Difco Laboratories) and 0.05 mg $ml^{-1}$ Deoxyribonuclease (DNase-I, Sigma Chemical Co.) The suspension was then given ten vigorous shakes to dislodge loosely attached cells and to disperse loosely adhering clumps of cells. Remaining clumps were removed by filtering the digest through 35 $\mu$m polyester mesh. The cell suspension was washed and resuspended in Hams F12 culture medium containing antibiotics and 17% donor calf serum. The cell suspension was counted using a haemocytometer, care being taken to distinguish between host cells and tumour cells (Stephens *et al.*, 1978). The cell yield from untreated tumours was usually in the range 1 to $8\times10^7$ cell $g^{-1}$.

In vitro *clonogenic cell survival assay*

The survival of tumour cells after either *in vivo* or *in vitro* drug treatment was assessed using the double-layer soft agar clonogenic assay developed by Courtenay (1976). The only modification was that red blood cells were not added to the culture medium, since they did not increase the cloning efficiency of caMT. The effect of *in vitro* drug treatment was expressed as surviving fraction but after *in vivo* treatment the fraction of surviving cells per tumour was used. This was calculated as the ratio of the number of colony-forming tumour cells in treated and untreated tumours. Points from all individual experiments have been included on the survival curves to give an indication of the variability and reproducibility of the data.

*Measurement of tumour growth delay*

A calibration curve technique was used to determine the size of tumours *in situ*. The tumour-bearing leg was passed through holes of known diameter in a perspex disc and the size of the tumour was taken to be the size of the largest hole through which the tumour would pass without resistance (Stephens *et al.*, 1984). This size was converted into tumour weight by comparison with a calibration curve which was constructed by measuring a number of tumours, dissecting them out and weighing them.

A minimum of six tumours per group were used in growth delay experiments. Tumours were measured every day and the time taken for each individual tumour to increase in weight by a factor of 4 was determined (T4×). Growth delay (GD) was then calculated as the difference in the median T4× of the treated and untreated groups.

*Karyotype analysis and DNA content*

Preparation of cells for karyotype was by standard techniques. Tumour cell suspensions were allowed to grow in monolayer for 18 h, exposed to colcemid (0.4 $\mu$g $ml^{-1}$, Gibco Diagnostics) for 2 h, harvested and incubated for 5 min in 0.075 M potassium chloride. Cells were fixed in methanol/glacial acetic acid (3:1) and dropped onto clean glass microscope slides. Slides were stained in 4% Giemsa stain (BDH Chemicals Ltd.) for chromosome counting. For DNA analysis cells were stained with ethidium bromide using the method of Vindeløv (1977) and DNA content was measured with an Ortho Cytofluorograf 50H system (Ortho Instruments, Mass, USA).

### Results

*Development of drug-resistance*

In order to develop drug resistance, caMT was treated in consecutive passages with single doses of melphalan, CY or *cis*-Pt. At each tumour passage, the first treated tumour to reach a size of four times its weight at the time of treatment was used to implant tumours for the next passage. Each agent was administered at the maximum dose which did not kill any mice (12 mg $kg^{-1}$ melphalan, 10 mg $kg^{-1}$ *cis*-Pt and 180 mg $kg^{-1}$ CY). CY was also used at 60 mg $kg^{-1}$ for some experiments.

Figure 1 shows changes in growth delay produced by repeated drug treatments with each drug. In each case there was a gradual development of drug resistance. The GD produced by 12 mg kg$^{-1}$ melphalan dropped from 8.6 d to 2.1 d, a reduction factor of 4.1 (GD in previously untreated tumour/GD in treated tumour), after 16 treatments. Twenty treatments with 180 mg kg$^{-1}$ CY reduced the growth delay produced by this dose from 15.9 to 1.5 days, a factor of 10.6, and 19 treatments with 10 mg kg$^{-1}$ *cis*-Pt reduced the growth delay from 9.2 d to 2.4 d, a factor of 3.8.

To test the effect of dose on the rate of development of resistance an experiment was performed with CY at two dose levels. Treatments were again given at each passage. In one case each dose was 60 mg kg$^{-1}$ CY and in the other 180 mg kg$^{-1}$ CY was used. For each the GD produced by both 60 mg kg$^{-1}$ and 180 mg kg$^{-1}$ CY was measured at every passage (Figure 2).

**Figure 1** Development of drug-resistance. Growth delay produced by given doses during repeated treatment with (a) 12 mg kg$^{-1}$ melphalan; (b) 10 mg kg$^{-1}$ *cis*-platinum; (c) 180 mg kg$^{-1}$ (◇, □) and 60 mg kg$^{-1}$(●) cyclophosphamide.

**Figure 2** The effect of drug dose on the rate of resistance development. The growth delay produced by (a) 60 mg kg$^{-1}$ and (b) 180 mg kg$^{-1}$ cyclophosphamide during repeated treatment with either 60 mg kg$^{-1}$ (--◆--) or 180 mg kg$^{-1}$ (—□—) cyclophosphamide at each passage.

The results show that treatment with a higher dose of drug does not bring about drug resistance more quickly than a lower dose. This was unexpected since, if resistance development was due to the selection of a pre-existing highly drug-resistant sub-population of tumour cells, the higher dose should eliminate sensitive cells more quickly and bring about a more rapid loss of tumour response.

### *Clonogenic cell survival after* in vivo *treatment*

The results of cell-survival studies on the lines which had received multiple drug treatments are given in Figure 3. The dose-survival curve for the line which had received 16 melphalan treatments (MTME16) measured 18 h after treatment is exponential through the origin and has a $D_{10}$ (dose to reduce survival by one log) of 7.8 mg kg$^{-1}$, which compares with 2.6 mg kg$^{-1}$ for wild-type caMT. This indicates a resistance factor ($D_{10}$ of resistant tumour/$D_{10}$ of sensitive tumour) of 3. This difference in slope was statistically significant ($P<0.01$) when the curves fitted by linear regression were compared using a *t* test.

**Figure 3** Dose-survival curves after *in vivo* treatment for lines which had received (a) 8 (MTME8, □) or 16 (MTME16, ●) treatments with $12\,mg\,kg^{-1}$ melphalan; (b) 20 treatments with $10\,mg\,kg^{-1}$ *cis*-platinum (MTCP20, ●) or (c) 16 treatments with $180\,mg\,kg^{-1}$ cyclophosphamide (MTCY16, ●). Curves for wild-type caMT are given for each drug (◇).

For the tumour which had received 8 previous treatments with melphalan the melphalan dose-survival curve had a $D_{10}$ of $5.6\,mg\,kg^{-1}$. A negative intercept ($n = 0.78$) was suggested when the data were fitted by least squares regression analysis but the errors on the fitted curve were such that the extrapolation number was not significantly different from one. Since the $D_{10}$ value of this curve is less than that seen after 16 previous treatments, a simple selective process on a pre-existing drug-resistant sub-population may not fully explain the development of resistance in this case.

Clonogenic cell survival assays performed on tumours which had received 20 treatments with *cis*-Pt (MTCP20) indicated that a change in the slope of the dose survival curve had accompanied the fall in growth delay. The $D_{10}$ increased from $2.9\,mg\,kg^{-1}$ to $5.25\,mg\,kg^{-1}$ (Figure 3b).

The *in vivo* dose-survival curve for MTCY16, which had received 16 treatments with CY is given in Figure 3c. A $D_{10}$ of $57\,mg\,kg^{-1}$ and an extrapolation number of 1.2 compares with $14.9\,mg\,kg^{-1}$ and 3.3 for the untreated caMT. There was therefore a significant decrease in the slope of the curve ($P < 0.001$, *t* test).

Clonogenic cell survival assays were also performed at each passage during repeated treatment with $60\,mg\,kg^{-1}$ CY. Table I shows the parameters of the survival curves. The tumour showed little response to $60\,mg\,kg^{-1}$ CY after 5 treatment passages according to a growth delay endpoint (Figures 1c, 2a) and this acquired resistance was associated with changes in the shape of the survival curve ($D_{10}$ increased from 14.9 to

**Table I** Parameters of cyclophosphamide dose-survival curves during repeated *in vivo* treatment with $60\,mg\,kg^{-1}$ cyclophosphamide. (Figures in brackets are 95% confidence limits)

| *Number of previous treatments* | *Survival curve parameters* | |
|---|---|---|
| | $D_{10}$*(mg kg⁻¹)* | *n* |
| 0 | 14.9 (13.2–17.1) | 2.6 (1.2–5.4) |
| 1 | 21.7 (17.2–29.3) | 5.6 (1.7–18) |
| 2 | 15.1 (11.2–23.4) | 15.0 (1.9–122) |
| 3 | 27.7 (19.0–50.9) | 3.0 (0.9–10.4) |
| 4 | 35.1 (26.3–52.8) | 1.6 (0.7–3.8) |
| 5 | 35.4 (26.7–52.7) | 1.3 (1.6–2.9) |

35.4 mg/kg$^{-1}$). The extrapolation numbers were poorly defined by the data and the trends in this parameter were not significant.

*Clonogenic survival after* in vitro *treatment*

The results of clonogenic cell survival assays following *in vivo* treatment can be influenced by factors other than inherent cellular sensitivity. However, some of these problems can be overcome by treating cells *in vitro* for 1 h and when this was done significant differences were again seen between the wild-type caMT and the drug resistant lines. Figure 4a shows that MTME16 had a $D_{10}$ of 1.9 $\mu$g ml$^{-1}$ compared with 0.63 $\mu$g ml$^{-1}$ for caMT ($P<0.001$, *t* test) and the resistant line also has a small shoulder on the curve (extrapolation number = 1.5).

The *in vitro cis*-Pt dose-survival curve for MTCP20 is shown in Figure 4b. There appears to be a change in slope ($D_{10}$ value of 4.7 $\mu$g ml$^{-1}$ compared with 3.1 $\mu$g ml$^{-1}$ for wild-type caMT) and a slight increase in the size of the shoulder (extrapolation number = 1.5 compared to 1 for wild-type caMT).

*Karyotype, DNA content and cell volume*

The distribution of chromosome numbers for the wild-type MT carcinoma was stable during one year of continuous passage *in vivo*. The modal chromosome number was 73 (range 50–80) and one metacentric marker chromosome was present in 95% of the cells. A slight drop in the modal chromosome number was detected for MTME16 and MTCP15 (modes of 69 and 68 respectively, Table II) and all of the metaphases analysed had a metacentric marker chromosome.

MTCY16 had fewer chromosomes than the wild-type MT carcinoma, with a mode at 63/64 chromosomes and a range of 36–154. A sub-metacentric marker chromosome was present in 70% of the cells. This differed from wild-type caMT in which the marker was metacentric. When the cells of MTCY16 were analysed immediately after the cessation of treatment all cells had at least two double minutes, which have been shown to be associated with gene amplification in some cases. These double minutes were not present when MTCY16 had been passaged 24 times without treatment although the line retained resistance to CY.

Table II also shows that MT and MTME16 had identical DNA contents (Relative $G_1$ DNA content, RGD = 1.9) and although MTCP15 was lower than wild type caMT (RGD = 1.8) it was within the range of values for different samples of wild-type tumour (1.8–2.0). The DNA content for MTCY16,

**Figure 4** Dose-survival curves after *in vitro* treatment for lines which had received (a) 16 treatments with 12 mg kg$^{-1}$ melphalan (MTME16, ●) or (b) 20 treatments with 10 mg kg$^{-1}$ *cis*-platinum (MTCP20, ●). Curves for wild-type caMT are given for each drug (◇).

however, appeared to be significantly lower than wild-type caMT (RGD = 1.6).

The peak cell volume for wild-type caMT and the three drug resistant lines were measured with a Coulter counter and pulse height analyser.

**Table II** Properties of drug-resistant lines of caMT

| | *Wild-type* | *ME16* | *CY16* | *CP15* |
|---|---|---|---|---|
| DNA content (RGD)[a] | 1.9 | 1.9 | 1.6 | 1.8 |
| Modal chromosome number | 73 | 69 | 63 | 68 |
| Peak cell volume ($\mu m^3$) | 1075 | 1061 | 918 | 1026 |
| Tumour volume doubling time[b] (days) | 0.8 | 0.9 | 0.9 | 0.9 |

[a]RGD = Relative $G_1$ DNA content (ratio of $G_1$ peaks of tumour cells and host cells); [b]Calculated from time to grow from 0.1 to 0.5 g.

MTCY16 was the only line which differed significantly ($P<0.01$, Mann–Whitney U test) from wild type MT carcinoma, and this had a 15% reduction in the mean peak cell volume (Table II). This was consistent with the reduction in DNA content and chromosome number seen in MTCY16.

*Tumour growth rate*

Although the volume doubling time values for the resistant lines were greater than for wild-type caMT (Table II) these differences were not significant (Mann–Whitney U test).

*The stability of drug resistance*

The growth delay produced by 12 mg kg$^{-1}$ melphalan in MTME16 did not change significantly in 29 untreated passages. However, both MTCP15 and MTCY16 increased in sensitivity in the first few passages after treatment was stopped. The growth delay produced by 10 mg kg$^{-1}$ *cis*-platinum increased from 0.2 d to 4.9 d in the first 6 passages of MTCP15 but it was still 4.1 d after 12 untreated passages compared with 9.2 d for the wild-type tumour. Similarly for MTCY16 the growth delay produced by 180 mg kg$^{-1}$ cyclophosphamide increased from 1.5 to 6.8 d after 7 untreated passages but was 6.7 d after 24 passages, still a significant reduction from the 15.9 d growth delay seen in the wild-type caMT.

*Cross-resistance studies*

Cross-resistance patterns in the drug-resistant lines were studied by *in vitro* treatment followed by clonogenic cell survival estimation (Table III). Cross-resistance between *cis*-Pt and melphalan was evident in both the *cis*-Pt-resistant (MTCP15) and melphalan-resistant (MTME16) lines. Likewise, there was an increase in the $D_{10}$ value of the *cis*-platinum dose-survival curve in the CY-resistant line (MTCY16). Thus these results confirm the general finding that *cis*-Pt is cross-resistant with alkylating agents.

MTCY16 showed a decreased sensitivity to melphalan, as would be expected from the similar modes of action of these two drugs. In MTME16 the growth delay produced by 180 mg kg$^{-1}$ CY (13 d) was slightly lower than that in wild-type caMT (15 d). Although this difference was small it was noted that when the melphalan-resistant line was repeatedly treated with 180 mg kg$^{-1}$ CY, resistance to CY developed more quickly than in

**Table III** Cross-resistance studies. Slopes of dose-survival curves following *in vitro* treatment ($D_{10}$ for drugs in $\mu g\,ml^{-1}$ and Do in Gy for $\gamma$-radiation). Values in brackets give ratio of slopes of curves for resistant line and wild-type caMT

| *Tumour line* | *Melphalan* | cis-*platinum* | *VP16* | *Bleomycin* | *Vindesine* | *CBDCA* | *γ-ray* |
|---|---|---|---|---|---|---|---|
| Wild-type | 0.63 | 3.1 | 8.6 | 22 | 2.5 | 109 | 1.5 |
| ME16 | 1.9<br>(3.0) | 5.7<br>(1.8) | — | — | — | — | 1.4<br>(0.93) |
| CP15 | 1.1<br>(1.7) | 4.7<br>(1.5) | 8.1<br>(0.94) | 22<br>(1.0) | 3.8<br>(1.52) | 286<br>(2.6) | 1.5<br>(1.0) |
| CY16 | 1.04<br>(1.65) | 5.8<br>(1.9) | — | — | — | — | 1.3<br>(0.87) |

wild-type caMT (Figure 5). After 4 treatments, for example, the growth delays for the two experiments with MTME16 were 4.3 and 7.9 d while in three series with caMT it was 14.6, 9.9 and 13.3 d. Resistance to melphalan was still evident after 8 treatments with CY.

VP16 and bleomycin have been used in combination with *cis*-Pt in the treatment of some tumours (Kelson *et al.*, 1978; Sierocki *et al.*, 1979) and the lack of cross-resistance between *cis*-Pt and these drugs in MTCP15 (Table III) supports this use. However, vindesine, which was shown by Seeber *et al.* (1982) to demonstrate collateral sensitivity in a *cis*-Pt resistant tumour line, demonstrated cross-resistance with *cis*-Pt in MTCP15.

CBDCA (*cis*-diammine-1, 1-cyclobutane dicarboxylato platinum II), an analogue of *cis*-platinum

**Figure 5** Development of resistance to 180 mg kg$^{-1}$ cyclophosphamide in the melphalan-resistant line of caMT (MTME16, ◇ and □ are two independent experiments). The dashed line indicates the development of cyclophosphamide-resistance in wild-type caMT from **Figure 1c.**

**Figure 6** Actual development of resistance to melphalan in caMT (○) compared with a two-compartment model involving sensitive cells plus a completely resistant sub-population of cells (---) or a partially resistant sub-population (—). Calculations were based on $D_{10}$ values of 2.6 mg kg$^{-1}$, 7.8 mg kg$^{-1}$ and infinity for the sensitive, partially resistant and completely resistant tumour cells.

which has been reported to be less toxic than *cis*-Pt (Harrap *et al.*, 1980), was also less effective in the *cis*-Pt resistant line than in wild-type caMT.

None of the drug-resistant lines showed any significant change in the response to γ-radiation.

### Discussion

The acquisition of drug-resistance in tumours is an important clinical problem which is still far from being fully understood. In this paper we have examined the development of resistance to three commonly used antitumour agents, melphalan, *cis*-platinum and cyclophosphamide in a murine mammary carcinoma and have found that existing models of drug-resistance development do not adequately explain the results obtained.

Repeated high dose drug treatment was found to bring about a reduction in tumour response as assayed by growth delay and clonogenic cell survival after *in vivo* and *in vitro* drug treatment. The degree of resistance achieved varied according to how this parameter was defined. For example, the growth delay induced by 12 mg kg$^{-1}$ melphalan was reduced by a factor of 4.1, while the ratio of the slopes of the dose-survival curves after clonogenic cell assay was 3.0 after both *in vivo* and *in vitro* treatment. This may reflect non-linearity in the dose-response curves and emphasizes the importance of using more than one assay to define therapeutic response.

Gross karyotypic differences between drug-sensitive and resistant tumour lines are a common finding (Parsons & Morrison, 1978; Peacock *et al.*, 1982; Tew *et al.*, 1983). In caMT there was a tendency for a lower modal chromosome number in the drug resistant lines. In the case of the cyclophosphamide-resistant line (MTCY16) this difference was large and was accompanied by a significant reduction in DNA content and cell size. Whether this is directly related to the drug-resistant phenotype is not known. The presence of double minute chromosomes in MTCY16 suggests that some genes may have been amplified since double minutes in cell populations resistant to methotrexate have been shown to reflect gene amplification (Schimke *et al.*, 1978). There is no documented evidence of gene amplification in alkylating agent-resistant cells, although Tew *et al.* (1983) did find double minutes in an alkylating agent-resistant subline of the Walker 256 breast carcinoma cell line. Double-minutes are often lost at mitosis when the selection pressure of drug treatment is removed (Kaufman *et al.*, 1979). This seems to have occurred here because after 24 untreated passages of MTCY16 the double minutes were no longer present. However, since at this time the line was still much more resistant than the wild-type tumour it would seem that double minutes were not uniquely associated with cyclophosphamide resistance. Alterations in the chromosome number distribution and in the marker chromosome were stable after cessation of drug treatment but it is not known how these might relate to cyclophosphamide resistance.

Analysis of the rate of tumour volume increase indicated that resistance to the three drugs used in this study was not due to changes in the growth rate during drug treatment. This confirms the findings of studies on other alkylating agent-resistant lines (Parsons & Morrison, 1978; Ball et al., 1966).

It was observed that melphalan resistance in MTME16 was stable when assayed using growth delay during passage without drug treatment. However, in MTCY16 and MTCP15 stability of resistance was only found after partial return of sensitivity, suggesting that perhaps two mechanisms, one stable and the other unstable, were involved in resistance in these two lines. Stable genomic changes are therefore likely to have been responsible for at least part of the resistance observed following treatment with the three drugs studied here. This is consistent with some reports of the stability of resistance to alkylating agents (D'Incalci et al., 1983; Frondoza et al., 1982; Schabel et al., 1978) although it is not a universal phenomenon since in other systems the resistance phenotype was found to be highly unstable (Berman & Steel, 1984).

Patterns of cross-resistance often show inconsistencies between different tumour systems, which makes their interpretation very difficult. This is demonstrated here by the observation of cross-resistance between *cis*-Pt and vindesine, when the only previous study on these two drugs showed collateral sensitivity (Seeber et al., 1982). The increased rate of CY-resistance development in MTME16 may be a reflection of such variations within a single tumour. Figure 5 suggests that the majority of the cells in MTME16 were not resistant to CY but a CY-resistant cell population could be rapidly selected by repeated CY treatment. This emergence of resistance to a second drug may be termed 'tertiary resistance' and it could be a serious limiting factor to the success of salvage chemotherapy in which a new drug is used following relapse during the course of the treatment of first choice. Even if no cross-resistance is observed initially when the treatment is altered, tumour responsiveness could rapidly decline during repeated treatment.

Current theories of drug resistance development are based on a two-compartment model in which the drug sensitive cells which compose the majority of the tumour are eliminated by drug treatment, leaving a pre-existing highly drug-resistant cell population to predominate. Skipper et al. (1978) produced an equation which enables the calculation of the number of treatments required to bring about complete drug resistance (which they define as being when the resistant and sensitive subpopulations are equal in size) if one knows the sensitivity of the sensitive and resistant cells and the growth kinetics of the two sub-populations. The individual treatments used in the development of resistance to melphalan, CY and *cis*-Pt in the MT carcinoma produced at least three decades of cell kill and the final resistant populations did not differ from the parent caMT in their growth rate. Therefore, since one might expect drug resistant cells to be present at a proportion of 1 in $10^5$ to 1 in $10^8$ (Skipper et al., 1978) we would have expected total resistance within 2–3 treatments if a completely resistant sub-population of cells was being selected. The slow rate of resistance development seen in this system is inconsistent with this simple model (see broken line in Figure 6).

One factor which could influence these results is the degree of resistance of the 'resistant' population. In all of the reports of drug resistance that we are aware of, resistance was not complete even after many treatments. This was recognised by Skipper et al. (1978) who included a 'sensitivity factor' for resistant cells in their equation to calculate the rate of resistance development. However, to simplify calculations total resistance was generally assumed.

By including a factor to take into account partial drug resistance, the loss of therapeutic response would be expected to occur more slowly. For example, the development of resistance to melphalan in caMT produced a line which had a 3-fold decrease in the slope of the dose-survival curve. If it is assumed that the sensitivity at the end of the series of treatments was equal to the sensitivity of the original resistant population, then the reduction in the growth delay might be expected to decrease as indicated by the full line in Figure 6. The appearance of resistance is delayed and the curve falls to a plateau whose height is determined by the sensitivity of the resistant sub-population. The resulting theoretical curve approximates more closely to the actual data than when complete drug-resistance is assumed, but is still not an ideal fit.

Since at the beginning of treatment the number of resistant cells is likely to be low, the recognition that the resistant cells have a finite sensitivity to treatment raises the possibility of total extinction of the resistant population. If drug resistant cells occur at a level of 1 in $10^6$–$10^8$, in a small tumour of $10^7$–$10^8$ cells, a cell kill of 1–2 logs of resistant cells may be sufficient to kill all resistant cells. The extinction of resistant cells may be an important factor limiting the emergence of MeCCNU resistance in the LL carcinoma (Stephens, unpublished results) as well as the drug resistance described here.

This idea could lead to the situation in which low doses of drug bring about drug-resistance more quickly than high doses. If a high dose of drug kills a large number of sensitive cells but also kills some 'resistant' cells, resistance may develop more slowly than at a lower dose which, although killing fewer sensitive cells, leaves more resistant cells in the tumour. This may be one reason why $180\,mg\,kg^{-1}$ CY did not bring about drug resistance more quickly than $60\,mg\,kg^{-1}$ CY in caMT.

Even a consideration of partial drug resistance, however, cannot fully explain the data presented for caMT. With any two-compartment model of drug resistance the expected change in the clonogenic survival curves would be the gradual elevation of a resistant 'tail'. Curves with slopes between the initial wild-type caMT and the final resistant lines were observed in caMT which would suggest that during the course of resistance development the tumours were composed largely of cells with intermediate sensitivities. Therefore, to think of a tumour simply in terms of two subpopulations, one sensitive and one resistant, may be misleading.

One further factor which may have some bearing on the rate of resistance development during treatment with high or low doses is the mutagenicity of many anti-cancer drugs, including the three used here. It is widely accepted that low doses of a cytotoxic mutagen could induce more mutants than higher doses because the relationship between the number of surviving mutants and dose of mutagen is often a bell-shaped curve. At higher doses the number of mutants observed falls off largely because of the increased cell kill. This has been seen for both physical and chemical mutagens in a variety of test systems (Major & Mole, 1978; Venitt *et al.*, 1984).

Thus, repeated treatments with low drug doses may result in a larger drug-resistant population than high doses of drug. This induction of drug resistant mutants is a further factor which is not considered in existing models describing the development of drug resistance but it is one which may be very important in multiple drug treatment regimes or when multiple treatments with a single drug are given.

Therefore, the data presented suggest that a model involving the selection of a pre-existing highly drug-resistant subpopulation is not adequate. An improvement is made when partial drug-resistance is taken into consideration but this is still not sufficient to fully explain the data. It would seem therefore that an alternative model for drug-resistance development is required possibly based on the broad spectrum of drug-sensitivities which we and others have observed in sub-lines derived from single tumours.

We thank Professor M.J. Peckham for his support during this work and Mr J.H. Peacock for useful discussions. We are also grateful to Mr A. Payne and Dr M. Ormerod for performing flow cytometry. T.J. McMillan acknowledges the financial support of the Cancer Research Campaign.

### References

BALL, C.R., CONNORS, T.A., DOUBLE, J.A., UJHAZY, V. & WHISSON, M.E. (1966). Comparison of nitrogen-mustard-sensitive and -resistant Yoshida sarcomas. *Int. J. Cancer*, **1**, 319.

BERGSAGEL, D.E., COWAN, D.H. & HASSELBACK, R. (1972). Plasma cell myeloma: response of melphalan-resistant patients to high-dose intermittent cyclophosphamide. *J. Can. Med. Assoc.*, **107**, 851.

BERMAN, R. & STEEL, G.G. (1984). Induced and inherent resistance to alkylating agents in human small-cell bronchial carcinoma xenografts. *Br. J. Cancer*, **49**, 431.

BROUWER, M., SMETS, L.A. & JONGSMA, A.P.M. (1983). Isolation and characterization of sublones of L1210 murine leukaemia with different sensitivities to various cytotoxic agents. *Cancer Res.*, **43**, 2884.

CLEMENTS, G.B. (1975). Selection of biochemically variant, in some cases mutant, mammalian cells in culture. *Adv. Cancer Res.*, **21**, 273.

COURTENAY, V.D. (1976). A soft agar colony assay for Lewis lung tumour and B16 melanoma taken directly from the mouse. *Br. J. Cancer*, **34**, 39.

D'INCALCI, M., TORTI, L., DAMIA, G., ERBA, E., MORASCA, L. & GARATTINI, S. (1983). Ovarian reticular cell sarcoma of the mouse (M5076) made resistant to cyclophosphamide. *Cancer Res.*, **43**, 5674.

FRONDOZA, C.G., TRIVEDI, S.M. & HUMPHREY, R.L. (1982). Development and characterisation of a cyclophosphamide-resistant mouse plasmacytoma cell line. *Cancer Treat. Rep.*, **66**, 1535.

GOLDIE, J.H. & COLDMAN, A.J. (1979). A mathematic model for relating the drug sensitivity of tumours to their spontaneous mutation rate. *Cancer Treat. Rep.*, **63**, 1727.

HARRAP, K.R. & 7 others. (1980). Antitumor, toxic and biochemical properties of cisplatin and eight other platinum complexes. In *Cisplatin. Current Status and New Developments*, Prestayko, *et al.* (eds) p. 193. Academic Press: New York.

HEPPNER, G.H., DEXTER, D.L., DENUCCI, T., MILLER, F.R. & CALABRESI, P. (1978). Heterogeneity in drug sensitivity among tumor cell subpopulations of a single mammary tumor. *Cancer Res.*, **38**, 3758.

KAUFMAN, R.J., BROWN, P.C. & SCHIMKE, R.T. (1979). Amplified dihydrofolate reductase genes in unstably methotrexate-resistant cells are associated with double minute chromosomes. *Proc. Natl Acad. Sci.* (*USA*), **11**, 5669.

KELSON, D.P., CRITOVIC, E., BAINS, M. & GOLBEY, R. (1978). Cis-diammine dichloroplatinum (II) (DDP) and bleomycin in the treatment of esophageal carcinoma. *Proc. Am. Assoc. Cancer Res.*, **19**, 352 (Abstract).

MAJOR, I.R. & MOLE, R.H. (1978). Myeloid leukaemia in X-ray irradiated CBA mice. *Nature*, **272**, 455.

PARSONS, P.G. & MORRISON, L. (1978). Melphalan-induced chromosome damage in sensitive and resistant human melanoma cell lines. *Int. J. Cancer*, **21**, 438.

PEACOCK, J.H., CASEY, G., McMILLAN, T.J. & STEPHENS, T.C. (1982). Chromosome damage in Lewis lung (LL) tumour lines with a spectrum of sensitivities to MeCCNU. *Br. J. Cancer*, **46**, 503.

PREISLER, H.D. (1982). Treatment failure in AML. *Blood Cells*, **8**, 585.

SCHABEL, F.M., TRADER, M.W., LASTER, W.R., WHEELER, G.P. & WITT, M.H. (1978). Patterns of resistance and therapeutic synergism among alkylating agents. *Antibiot. Chemother.*, **23**, 200.

SCHIMKE, P.R., KAUFMANN, R.J., ALT, F.W. & KELLEMS, R.F. (1978). Gene amplification and drug resistance in cultured murine cells. *Science*, **202**, 1051.

SCHMID, F.A., OTTER, G.M. & STOCK, C.C. (1980). Resistance patterns of Walker carcinoma 256 and other rodent tumors to cyclophosphamide and L-phenylalanine mustard. *Cancer Res.*, **40**, 830.

SEEBER, S., OSIEKA, R., SCHMIDT, C.G., ACHTERRATH, W. & CROOKE, S.T. (1982). *In vivo* resistance towards anthracylines, etoposide and cis-diamminedichloroplatinum (II). *Cancer Res.*, **42**, 4719.

SIEROCKI, J.S. & 6 others. (1979). Cis-dichlorodiammine platinum (II) and VP-16-213: An active induction regimen for small cells carcinoma of the lung. *Cancer Treat. Rep.*, **63**, 1593.

SKIPPER, H.E., SCHABEL, F.M. & LLOYD, H.H. (1978). Experimental therapeutics and kinetics: Selection and overgrowth of specifically and permanently drug-resistant tumour cells. *Semin. Hematol.*, **15**, 207.

STEPHENS, T.C., ADAMS, K. & PEACOCK, J.H. (1984). Identification of a subpopulation of MeCCNU resistant cells in previously untreated Lewis lung tumours. *Br. J. Cancer*, **50**, 77.

STEPHENS, T.C., CURRIE, G.A. & PEACOCK, J.H. (1978). Repopulation of $\gamma$-irradiated Lewis lung carcinoma by malignant cells and host macrophage progenitors. *Br. J. Cancer*, **38**, 573.

STEPHENS, T.C. & PEACOCK, J.H. (1982). Clonal variation in the sensitivity of B16 melanoma to m-AMSA. *Br. J. Cancer*, **45**, 821.

STEPHENS, T.C., PEACOCK, J.H. & SHELDON, P.W. (1980). Influence of in vitro assay conditions on the assessment of radiobiological parameters of the MT tumour. *Br. J. Radiol.*, **53**, 1182.

TEW, K.D., MOY, B.C. & HARTLEY-ASP, B. (1983). Acquired drug-resistance is accompanied by modification in the karyotype and nuclear matrix of a rat carcinoma cell line. *Exp. Cell. Res.*, **149**, 443.

VENITT, S., CROFTON-SLEIGH, C. & FORSTER, R. (1984). Bacterial mutation assays using reverse mutation. In *Mutagenicity Testing: A Practical Approach*, Vennit, S. & Parry J.M. (eds) p. 81. IRL Press: Oxford.

VINDELØV, L.L. (1977). Flow microfluorometric analysis of nuclear DNA in cells from solid tumors and cell suspensions. *Virchows. Arch. B. Cell. Path.*, **24**, 227.